# Modified prolonged exposure therapy as Early Intervention after Rape (The EIR-study): study protocol for a multicenter randomized add-on superiority trial

**DOI:** 10.1186/s13063-023-07147-w

**Published:** 2023-02-21

**Authors:** Tina Haugen, Joar Øveraas Halvorsen, Oddgeir Friborg, Melanie Rae Simpson, Paul Jarle Mork, Gustav Mikkelsen, Ask Elklit, Barbara O. Rothbaum, Berit Schei, Cecilie Hagemann

**Affiliations:** 1 Department of Psychology, Norwegian University of Science and Technology (NTNU), NO-7491 Trondheim, Norway; 2 Department of Clinical and Molecular Medicine, Norwegian University of Science and Technology (NTNU), NO-7491 Trondheim, Norway; 3 St. Olavs Hospital, Trondheim University Hospital, Pb. 3250 Torgarden, 7006 Trondheim, Norway; 4 Department of Psychology, The Arctic University of Norway (UiT), Pb. 6050 Langnes, N-9037 Tromsø, Norway; 5 Department of Public Health and Nursing, Norwegian University of Science and Technology (NTNU), Pb. 8905, N-7491 Trondheim, Norway; 6 Department of Clinical Chemistry, St. Olavs Hospital, Trondheim University Hospital, Pb. 3250 Torgarden, 7006 Trondheim, Norway; 7 National Danish center for Psychotraumatology, University of Southern Denmark, Campusvej 55, 5230 Odense, Denmark; 8 Department of Psychiatry, Veterans Program and the Trauma and Anxiety Recovery Program, Emory University School of Medicine, Atlanta, USA; 9 Department of Obstetrics and Gynecology, St. Olavs Hospital, Trondheim University Hospital, Pb. 3250 Sluppen, NO-7006 Trondheim, Norway

## Abstract

**Background:**

Sexual assault and rape are the traumatic life events with the highest probability for posttraumatic stress disorder (PTSD), which can have devastating consequences for those afflicted by the condition. Studies indicate that modified prolonged exposure (mPE) therapy may be effective in preventing the development of PTSD in recently traumatized individuals, and especially for people who have experienced sexual assault. If a brief, manualized early intervention can prevent or reduce post-traumatic symptoms in women who have recently experienced rape, healthcare services targeted for these populations (i.e., sexual assault centers, SACs) should consider implementing such interventions as part of routine care.

**Methods/design:**

This is a multicenter randomized controlled add-on superiority trial that enrolls patients attending sexual assault centers within 72 h after rape or attempted rape. The objective is to assess whether mPE shortly after rape can prevent the development of post-traumatic stress symptoms. Patients will be randomized to either mPE plus treatment as usual (TAU) or TAU alone. The primary outcome is the development of post-traumatic stress symptoms 3 months after trauma. Secondary outcomes will be symptoms of depression, sleep difficulties, pelvic floor hyperactivity, and sexual dysfunction. The first 22 subjects will constitute an internal pilot trial to test acceptance of the intervention and feasibility of the assessment battery.

**Discussion:**

This study will guide further research and clinical initiatives for implementing strategies for preventing post-traumatic stress symptoms after rape and provide new knowledge about which women may benefit the most from such initiatives and for revising existing treatment guidelines within this area.

**Trial registration:**

ClinicalTrials.gov NCT05489133. Registered on 3 August 2022

**Supplementary Information:**

The online version contains supplementary material available at 10.1186/s13063-023-07147-w.

## Administrative information

**Table:** 

**Title**	Modified prolonged exposure therapy as an Early Intervention after Rape (The EIR-study): Study protocol for a multicenter randomized add-on superiority trial.
**Trial registration**	The trial has been registered in the clinicaltrials.gov (ClinicalTrials.gov Identifier: NCT05489133) Aug 3, 2022.
**Protocol version**	Protocol version 2.0 is currently active.
**Funding**	The EIR-study has received funding from the Research Council of Norway (project #320637), the Department of Clinical and Molecular Medicine, the Faculty of Medicine and Health Sciences, Norwegian University of Science and Technology (NTNU), and from the Department of Obstetrics and Gynecology, St. Olavs hospital, Trondheim University Hospital. The non-governmental organization Norwegian Women's Public Health Association (N.K.S.) is a collaborator in the project.
**Roles and responsibilities: contributorship**	Names, affiliations, and roles of protocol contributors: **Cecilie Hagemann,** Principal Investigator. Department of Clinical and Molecular Medicine, Norwegian University of Science and Technology (NTNU). Department of Obstetrics and Gynecology, St. Olavs hospital, Trondheim University Hospital. cecilie.hagemann@ntnu.no **Joar Øveraas Halvorsen,** Co-Principal Investigator. Department of Psychology, Norwegian University of Science and Technology (NTNU). St. Olavs hospital, Trondheim University Hospital. joar.halvorsen@ntnu.no **Berit Schei,** Associate Investigator. Department of Public Health and Nursing, Norwegian University of Science and Technology (NTNU). Department of Obstetrics and Gynecology, St. Olavs hospital, Trondheim University Hospital. berit.schei@ntnu.no **Tina Haugen,** Associate Investigator, PhD candidate. Department psychology and Department of Clinical and Molecular Medicine, Norwegian University of Science and Technology (NTNU). St. Olavs hospital, Trondheim University Hospital. tina.haugen@ntnu.no **Oddgeir Friborg**, Associate Investigator. Department of Psychology, The Arctic University of Norway. (UiT). oddgeir.friborg@uit.no **Melanie Rae Simpson**, Trial statistician. Department of Public Health and Nursing, Norwegian University of Science and Technology (NTNU). melanie.simpson@ntnu.no **Paul Jarle Mork,** Associate Consultant. Department of Public Health and Nursing, Norwegian University of Science and Technology (NTNU). paul.mork@ntnu.no **Gustav Mikkelsen**, Associate Consultant. Department of clinical chemistry, St. Olavs hospital, Trondheim University Hospital. Department of Clinical and Molecular Medicine, Norwegian University of Science and Technology (NTNU). Gustav.mikkelsen@stolav.no **Ask Elklit,** Associate Consultant. National Center for Psychotraumatology, University of Southern Denmark. aelklit@health.sdu.dk **Barbara O. Rothbaum,** Associate Consultant. Department of Psychiatry and Behavioral Sciences, Emory University School of Medicine. brothba@emory.edu
**Name and contact information for the trial sponsor**	St Olavs hospital HF, Pb. 3250 Torgarden. 7006 Trondheim.
**Roles and responsibilities: sponsor and funder**	The sponsor covers indemnity insurances and legal liability for this study, and funds research infrastructure such as general, personnel expenses, data collecting systems and secure data storage. This study is an investigator-initiated study; hence, the public funding organization (Research Council of Norway) has no role in formulations of hypotheses and study design, data collection, analyses, interpretation of the data or in writing of the manuscript.

## Background

Although the reported prevalence varies between countries, rape and sexual assaults are a global problem. The National Intimate Partner and Sexual Violence Survey indicated that up to 20% of women and 7% of men in the USA had experienced rape or attempted rape during their life [1, 2]. The World Health Organization World Mental Health surveys estimated a prevalence of sexual assault among women to be between 1.8% (in Spain) and 26.1% (in the USA) [3, 4]. A systematic review on the global prevalence of sexual assault found that across 22 studies, past-year prevalence of sexual assaults ranged from 0% (in China) to 59.2% (in Turkey) for women and 0.3 to 55.5% for men [5]. Another review found that the prevalence of completed sexual assault among female college students (while in college) ranged from 14.2 to 23.1% [6].

A meta-analysis addressing the relationship between trauma and psychiatric disorders concluded that those sexually assaulted are significantly more prone to develop mental health symptoms compared to those exposed to other traumas, notably post-traumatic stress disorder and depression [7]. Among the lifetime traumas a person may be subjected to, interpersonal trauma and especially rape are associated with a particularly high risk of PTSD [8]. In Sweden, researchers found that amongst 317 female victims of rape who sought help, 39% of the women developed PTSD within 6 months post-trauma, and 47% suffered from moderate depression [9].

The corollaries of trauma generally involve acute stress symptoms primarily characterized by intrusive memories and flashbacks, apprehension, and elevated psychophysiological activation [10]. These symptoms will for most individuals abate and diminish after 1–3 months through natural recovery processes [11]. Yet, for others, stress symptoms persist and may develop into PTSD, which increases the risk of other comorbid health issues such as mood or anxiety disorders [7], alcohol or substance abuse, and a higher risk for suicide [12–16].

Two recent systematic reviews and meta-analyses identified 17 published studies on different early interventions after sexual assault to prevent PTSD and other mental health disorders [17, 18]. The interventions included video interventions [19], psychoeducation and cognitive behavioral strategies [20], and EMDR [21]. The effect sizes in reducing post-traumatic stress symptoms were in the small-to moderate range [18]. A pilot study (*N* = 137) used modified prolonged exposure therapy in the emergency department for people who had suffered different types of traumas and found that those who received mPE early after trauma had a larger reduction in post-traumatic stress symptoms 12 weeks post-treatment compared to the control group consisting of assessment only. The study also found that the effect of mPE was larger for those who had experienced rape than for those who had experienced other types of trauma, for example motor vehicle accidents [22]. A more recent Swedish trial that replicated this study has shown similar results [23] indicating that mPE early after rape may hinder further aggravation of PTSD symptoms. This is a promising alley for acquiring more knowledge about how prevention trials should be designed or tailored.

The implementation of preventive measures following rape may stand at odds with existing international guidelines recommending active monitoring, or “watchful waiting” for someone who has experienced a potentially traumatizing event such as rape [24]. Active monitoring involves regular follow-up of persons showing signs of post-traumatic stress symptoms, but who do not fulfill the criteria for clinical intervention. The guidelines state that preventive measures are not recommended at group level during the first month. Yet, it seems like the first weeks or months can be decisive for the development of PTSD. According to the fifth edition of the Diagnostic and Statistical Manual of Mental Disorders (DSM-5) [25], a PTSD diagnosis is not implied until a minimum of 4 weeks has passed since the traumatic event. This, alongside the fact that PTSD has an identifiable starting point (the incident), can present a “window of opportunity” to intervene before the post-traumatic stress symptoms stabilize and become manifest. Several studies imply the existence of such transient opportunities in the aftermath of trauma [26–31]. It is hypothesized that the individual stress responses in the acute phase are involved in the processes of consolidation and retrieval of memory. Regarding the development of PTSD, this can be thought of as the time period when fear memories are “born”.

One hypothesis is that PTSD is caused by the failure of extinction. Extinction is a process of gradual reduction of a response to a conditioned stimulus when the stimulus is presented without reinforcement, and it is conceptualized as an active learning process in which newly acquired memories compete with or suppress the expression of the original fear memory. Fear is considered a normal response to trauma, and the majority of individuals who experience a traumatic event will find that the fear extinguishes over time. However, for a significant minority, the fear will not extinguish and may develop into PTSD, in which they feel haunted by the event. Therefore, early extinction training (e.g., exposure therapy) could have the potential to change the consolidation of the trauma memory. Exposure to trauma reminders in the absence of the anticipated negative consequences is hypothesized to extinguish conditioned emotional reactions [31]. The immediate period following a rape incidence may therefore represent an important opportunity for preventing PTSD that is missed while we are “watchfully waiting”.

Sexual and reproductive health may also be severely affected after rape, such as sexual dysfunction, chronic pelvic pain, and vulvar pain which are described after sexual assault [32, 33]. Both fear of sex, lack of desire and arousal, and orgasm problems have been reported, in addition to vaginismus [34]. A Dutch follow-up study of young rape victims treated for PTSD observed few differences between raped and non-traumatized women with regard to sexual activity [35]. However, rape victims had a higher risk of sexual dysfunctions (e.g., lubrication problems and pain) and pelvic floor muscular dysfunction compared to controls. Since hyperactive pelvic floor muscles may stem from chronic mental stress [36, 37], prevention of PTSD may be clinically important if it intersects the connection between sexual assaults and adverse outcomes such as dyspareunia and sexual dysfunctions [38], thus indicating a mediator mechanism [39]. The present study therefore also examines if prevention of PTSD also lowers pelvic floor hyperactivity and hence prevents the development of sexual problems.

The context for the present prevention trial is the Norwegian specialized sexual assault centers (SACs) that are established throughout Norway, which provide health care services to patients who have experienced sexual assaults [40]. Medical treatment, forensic examination, and psychological support and counseling are delivered around the clock by specialized physicians, nurses, and social workers. Most SACs in Norway lack psychological staffing, and nurses or social workers are responsible for the immediate psychosocial care in the aftermath of a sexual assault. The guidelines describing psychosocial support after rape are vague, resulting in different approaches at each center.

### Aims

The primary aim of the present trial is to investigate the effectiveness of an early modified prolonged exposure (mPE) intervention shortly after rape as a preventive measure of post-traumatic stress symptoms. This is a superiority trial, and our hypothesis is that the patients receiving mPE in addition to TAU will report fewer post-traumatic stress symptoms 3, 6, and 12 months post-trauma as compared to the TAU alone. We expect that the mPE is safe and can be offered shortly after rape at the SACs, and that it can be delivered by SAC nurses or social workers trained in PE.

Secondary aims are to explore whether mPE + TAU is more effective than TAU in preventing symptoms related to depression, anxiety, pelvic floor hyperactivity, and sexual dysfunction 3 months post-trauma, and after 6 and 12 months.

A mediator analysis will be conducted to examine if the post-traumatic stress symptom levels may represent a mediating mechanism for the connection between treatment type (mPE or TAU) and sexual health in terms of hyperactive pelvic floor and sexual dysfunction.

Furthermore, we will perform exploratory analyses to investigate whether certain baseline characteristics may modify or predict the effect of mPE and TAU, for instance socioeconomic status, characteristics related to the trauma event, prior traumatization, peritraumatic factors (e.g., tonic immobility, drug influence), hair cortisol reflecting levels before trauma, and protective factors (e.g., resilience). These variables will be included as moderators in the analyses to examine if any subgroups of women respond better or worse to the treatment. We will also examine if sleeping problems (e.g., self-reported insomnia), actigraphic patterns, and salivary cortisol excretion may play a moderating role.

## Methods

### Trial design

The present protocol is designed to deliver mPE or TAU to all women seeking one of three SACs. This is a two-armed parallel design which will randomly assign participants to either mPE + TAU or TAU alone following baseline assessments. The mPE participants will be offered the same medical treatment, forensic examination, and psychosocial follow-up options as the TAU group, with the mPE intervention provided as additional treatment. Outcome measures are collected at 6 weeks post-trauma and after 3, 6, and 12 months.

### Blinding

Due to the nature of the interventions, blinding of patients and therapists is not possible. Psychology students assessing for PTSD symptoms are blinded. Researchers analyzing study data and statisticians involved in the analysis will be blinded until the data collection of primary outcomes (3 months post-trauma) is completed. The data monitoring committee is also blinded.

In case of exceptionally severe adverse events, e.g., completed suicide, the data monitoring committee (DMC) will have access to information regarding treatment condition in order to consider whether the adverse event is likely related to the treatment and whether the trial should be stopped.

This trial adheres to the SPIRIT guidelines and methodology [41]; see Additional file 1 for the SPIRIT checklist.

### Research setting

Norway has 24 sexual assault centers (SACs) offering medical assessment and treatment, forensic documentation, and psychosocial support for patients 16 years of age or older who experience rape or attempted rape. Treatment is usually performed by physicians, nurses, and social workers. The mPE group will be assigned up to five weekly sessions of 60–90 min mPE in addition to TAU. The TAU-group will receive the psychosocial support and treatment that is available at each respective SAC. Three SACs will participate in this study: Trondheim, Oslo, and Vestfold. The SACs offered to participate in the study and were chosen based on their already well-organized service of psychosocial support, their size, and their geographic distribution. However, only three SACs agreed to participate. The research team has presented the study directly to eligible SAC’s staff leaders, as well as on the yearly national Norwegian SAC leader seminars.

### Eligibility criteria

Patients who seek help at one of the SACs within 72 h after an episode of rape or attempted rape are screened for eligibility. The definition of rape or attempted rape for this purpose is “penetration in any body orifice (by penis, finger, foreign body), but also attempted penetration leading to a sufficient mental reaction (helplessness, without control, intense fear”.

**Fig. 1:**
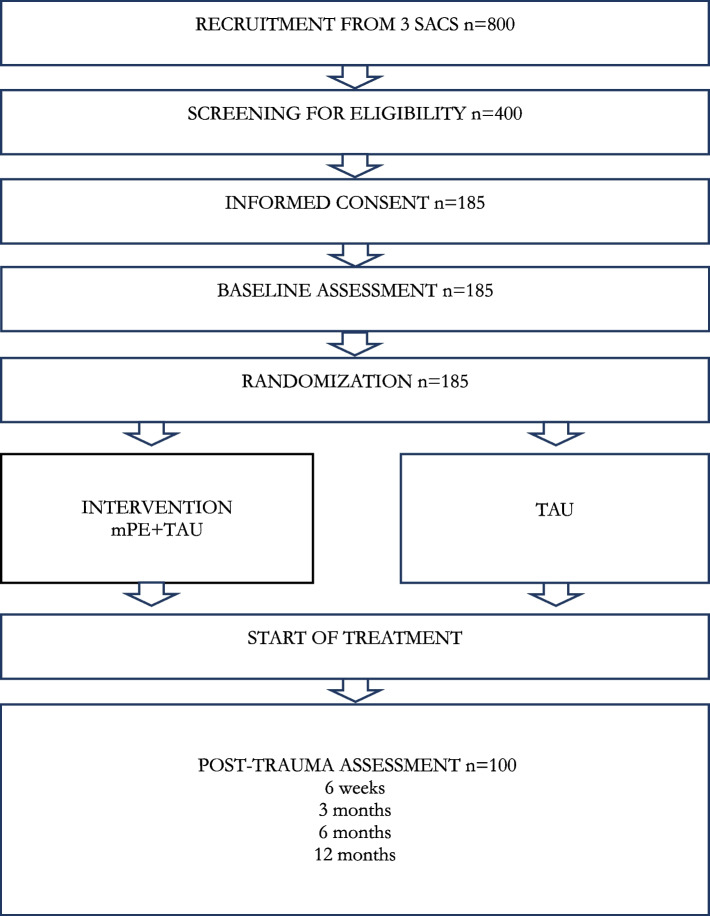
Flowchart of estimated patients included in the study

*Inclusion criteria* are female patients consenting to participate, being 16 years of age or older, having had at least one SAC consultation within 3 days after the rape/attempted rape, and being able to participate in the intervention within 14 days after the rape/attempted rape (Fig. 1).

*Exclusion criteria* are patients being younger than 16 years of age, of male gender, time since rape is more than 72 h, not being able to participate in the intervention within 14 days after the rape/attempted rape, being under ongoing threat of repeat violence, having cognitive disability, being acute psychotic and/or acute suicidal, having severe alcohol and/or drug abuse problem, currently receiving treatment for PTSD, not speaking Norwegian, and/or having total amnesia for the event.

### Recruitment

The initial assessment is conducted by senior staff members at the SACs following the first acute consultation (age, gender, type of assault, current psychiatric and medical status). Women who accept further psychosocial support as offered by a SAC employee are scheduled for a new meeting (on-site or by telephone) within a few days. Women that are eligible are invited to participate in the trial. Consenting women are then followed up by a research assistant (RA) who provides more detail about the study before providing a final written consent and proceeding to the electronic questionnaires for registering baseline data. The RA will at the same time provide instructions and equipment for collecting saliva samples for awakening cortisol response at home and attach accelerometers for monitoring physical activity and sleep. A hair sample will also be collected to extract cortisol levels that may indicate longer-term accumulation of this stress hormone. Finally, a trained clinical psychology student arranges for a clinical interview using the PTSD Symptom Scale Interview (PSS-I-5), either by phone or video conference.

### Randomization

Consenting patients will be randomly allocated 1:1 to mPE + TAU or TAU by the RA using a computer-generated allocation sequence (in web-CRF3, see below). The randomization is stratified on the SAC level using permuted blocks with random block sizes to ensure allocation concealment and comparable study arm sample sizes. A participant is deemed randomizable if critical baseline data have been collected, i.e., informed consent, the questionnaire, and clinical interview. For logistical reasons, consenting participants that have provided cortisol samples from hair and saliva and actigraphic recordings for a minimum of 3 days will be randomized.

### Intervention

#### Modified prolonged exposure therapy

In the mPE intervention group, patients will be assigned to a maximum of 5 weekly sessions of 60–90 min mPE (Table 1). mPE is based on the original PE treatment protocol developed by Foa, Hembree, Rothbaum, and Rauch [42, 43], and modifications are based on a study developed by Rothbaum and colleagues [22]. The main procedures in mPE are psychoeducation about normal reactions to trauma, information on which factors can maintain PTSD symptoms, and imaginal and in vivo exposure techniques and breathing training. The exposure focuses on the specific trauma memory and associated trauma reminders. In vivo exposure includes repeatedly confronting situations, activities, and objects that are unrealistically perceived as unsafe after the trauma. Imaginal exposure (e.g., talking through the trauma memory repeated times) is followed by thorough processing of thoughts and perspectives about the world and the self after the rape (e.g., guilt and self-blame). The mPE procedures are delivered in a systematic manner to enhance emotional engagement to the trauma memory, and to resist avoidant behavior to reduce stress. mPE sessions are audiotaped on a recorder device integrated in the smartphone application PE Coach [44], and participants are encouraged to listen to the recordings between sessions for repeated exposures.

**Table 1:** Schedule of enrollment, interventions, and assessments

	**Study period**						
	**Enrollment**	**Baseline**	**Allocation**	**Post-allocation**			
**Timepoint**				**Post-treatment (6 weeks)**	**3 months**	**6 months**	**12 months**
**Enrollment**							
Eligibility screen	X						
Informed consent	X						
Allocation			X				
**Interventions**							
Up to 5 sessions mPE + TAU			X				
TAU			X				
**Assessments**							
Demographics		X					
PSS-I-5 interview		X		X	X	X	X
PCL-5		X		X	X	X	X
Patient Health Questionnaire-9		X		X	X	X	X
General Anxiety Disorder-7		X		X	X	X	X
Quality of Life, mental aspects EQ-5D-5L		X		X	X	X	X
Bergen Insomnia Scale		X		X	X	X	X
Amsterdam Hyperactive Pelvic Floor Scale for Women		X		X	X	X	X
Female Sexual Function Index		X		X	X	X	X
Gynecological history		X					
Questionnaire on Chronic pelvic pain		X		X	X	X	X
The Stressful Life Events Screening Questionnaire		X		X	X	X	X
Tonic Immobility Scale		X					
Resilience Scale for Adults		X					
Questionnaire on medicinal and drug use		X		X	X	X	X
Accelerometry measurements		X		X	X	X	X
Saliva cortisol		X		X	X	X	X
Hair cortisol		X		X	X	X	X
AE/SAE monitoring				X	X	X	X

Nurses and social workers that conduct the mPE intervention will receive education about the theoretical underpinnings of PE, as well as hands-on training in the use of PE and the mPE protocol by an experienced PE-certified therapist and supervisor. Training consists of a mandatory 4-day workshop with lectures, which includes roleplaying the main procedures, self-studies, videos, and group discussions around methodological and practical implications of mPE. PE therapists will be supervised individually before and after their first mPE consultation and/or as needed. Audio recordings from mPE sessions will be used in group supervision.

#### Treatment as usual

The TAU intervention group will get the standard care that is routinely provided at the SACs. This may include psychosocial counseling, psychoeducation, and practical advice with a varying number of consultations, in addition to regular medical and/or forensic follow-up consultations by the SAC personnel. To reduce the risk of contamination, the nurses or social workers delivering TAU will not be trained in mPE.

### Adherence

All mPE sessions during the intervention period will be audiotaped to screen for adherence and fidelity using an adherence and competence scale developed for PE. A power estimation based on a two-way ANOVA mixed effect model, with two raters for each patient observation, indicated a need for 13 subjects in order to detect an intraclass correlation coefficient (ICC) of minimum 0.90 with a 95% lower confidence interval of 0.70.

Both mPE and TAU therapists must fill out web-based case report forms to describe which treatment components they included in the consultations, and to what extent they included components not part of the mPE procedure.

## Data collection and management

The primary assessment point will be at 3 months, whereas secondary assessment points will be 6 weeks and 6 and 12 months post-trauma. Patients’ self-reported questionnaire data are collected using an electronic survey system, administered by the Clinical Research Unit at St. Olavs University Hospital and NTNU. Participants will receive an email and SMS with links to the questionnaire after written consent. To access the questionnaire, respondents must use a secure login via BankID (a Norwegian Bank identifier code). At the first assessment, participants receive a survey package, consisting of information about the study, consent form, and the baseline questionnaire. For the following four assessments, only the questionnaire is sent out. For the questionnaire to be submitted, the PTSD symptom checklist (PCL-5) and the quality-of-life measure (EQ-5D-5L) are set as default.

We request consent for review of participants’ medical records and for the collection of saliva and hair samples to assess cortisol. Participants are asked to report sociodemographic and background data: the year of birth; height and weight; self-perceived gender identity; primary language; relationship and cohabitation status; educational and employment status; economic status; gynecological history (contraceptive use, previous pregnancies, menopausal status); sexual matters (having current sexual partner, sexual orientation, sexual satisfaction); questions on chronic pelvic and vulvar pain; use of prescription medication, alcohol, tobacco, and illicit drugs; and self-reported physical and mental health in general. The Tonic Immobility Scale is used at baseline to collect information about peritraumatic reactions during the rape and the Resilience Scale for adults as a measure for protective factors. The RA will promote participant retention.

## Assessments

### Primary outcome measures

#### PTSD symptom scale interview (PSS-I-5)

The PSS-I-5 is a 24-item semi-structured interview for assessing PTSD symptoms since the recent episode/in the past month and makes a diagnostic determination based on DSM-5 criteria for PTSD. The interview starts with a trauma screen to identify a criterion A trauma. Questions assess for frequency and intensity of 20 DSM-5 PTSD symptoms, and symptom items are rated on a 5-point scale of frequency and severity ranging from 0 (not at all) to 4 (6 or more times a week/ severe). Symptoms are considered present when rated 1 or higher. The sum of the 20 items yields a total PTSD symptom severity score, ranging from 0 to 80, with scores between 23 and 80 identifying a probable PTSD diagnosis. The PSSI-5 is a valid and reliable instrument for assessing PTSD diagnosis and severity [45]. We use a version that was translated into Norwegian with permission from the authors.

#### PTSD symptom checklist (PCL-5)

The PCL-5 is a 20-item self-report measure that assesses the 20 DSM-5 symptoms of PTSD. Items are rated on a 5-point Likert scale (0 = “not at all” to 4 = “extremely”), resulting in a total score between 0 and 80. Scores lower than 31–33 may indicate the patient either has subthreshold symptoms of PTSD or does not meet the criteria for PTSD. A total score of 31–33 or higher suggests the patient may meet the diagnostic criteria for PTSD. The PCL-5 has a variety of purposes, including monitoring symptom change during and after treatment, screening individuals for PTSD, and making a provisional PTSD diagnosis, and has demonstrated excellent reliability and validity in different trauma populations [46]. We use a translated Norwegian version.

### Secondary outcome measures

#### Patient Health Questionnaire (PHQ-9)

The PHQ-9 is based on the DSM-4 diagnostic criteria for major depressive disorder and has remained unchanged in the *DSM-*5 update. It uses nine items to assess and monitor the severity of depression symptoms during the last 2 weeks. Participants respond on a 4-point Likert scale (0—not at all, 1—several days, 2—more than half the days, and 3—nearly every day), yielding a total score range of 0–27 [47]. The PHQ-9 has also been shown to detect subthreshold depressive disorder in the general population [48]. We use a translated Norwegian version that has been validated in a large female sample [49].

#### General Anxiety Disorder (GAD-7)

The GAD-7 is a seven-item self-report measure used to describe the DSM-IV diagnostic criteria for generalized anxiety disorder. Participants are asked how often during the last 2 weeks they have encountered anxiety symptoms on a scale from 0 to 3 on a 4-point Likert-scale (0 = not at all, 1 = several days, 2 = more than half the days, and 3 = nearly every day), resulting in a total score between 0 and 21. The GAD-7 is valid for screening for GAD and assessing its severity in clinical practice [50]. We use a validated Norwegian-translated version of the GAD-7 [51].

#### Quality of life (EQ-5D-5L)

The EQ-5D-5L is a self-report measure for health-related quality of life questionnaire. The scale measures the quality of life on a 5-component scale including mobility, self-care, usual activities, pain/discomfort, and anxiety/depression. Each level is rated on a scale that describes the degree of problems in that area (i.e., I have no problems walking about, slight problems, moderate problems, severe problems, or unable to walk). EQ-5D-5L also has an overall health scale (the EQ VAS) where the rater selects a number between 1 and 100 to describe the condition of their health, 100 being the best imaginable [52]. We use a translated Norwegian version.

#### Bergen Insomnia Scale (BIS)

The BIS is a six-item self-report scale based on the DSM-4 diagnostic manual for assessing the number of days a week nocturnal symptom is present (i.e., sleep onset, maintenance, and early morning wakening insomnia) as well as daytime impairments (non-restorative sleep, daytime sleepiness, and dissatisfaction with sleep). The BIS may be used to decide if formal diagnostic inclusion criteria are satisfied (excluding the non-restorative sleep item), but in the present study, the week scores for nocturnal symptoms and daytime impairments are used to quantify reductions in these respective symptom load areas. The BIS is developed in Norway and validated on a Norwegian population [53].

#### Amsterdam Hyperactive Pelvic Floor Scale for Women (AHPFS-W)

The AHPFS-W is a self-report questionnaire to assess pelvic floor activity and pain [35]. The questionnaire includes 30 items on a 5-point Likert scale with scores varying from 1 (never) to 5 (very often). Twenty-five of the 30 items are categorized into six different subscales: vulvar pain symptoms, abdominal pain and defecation symptoms, micturition problems, urinary tract infection, rectal problems, and physiological symptoms of general stress/tension. A total score from 6 to 30 indicates the degree of pelvic floor hyperactivity, with scores from 13 to 30 indicating moderate to severe hyperactivity in the pelvic floor. We use a translated and validated Norwegian version [54] that is not yet published.

#### Female Sexual Function Index (FSFI)

The FSFI is a multidimensional self-report instrument for the assessment of female sexual function. It contains 19 items divided into six subscales including desire, subjective arousal, lubrication, orgasm, satisfaction, and pain. FSFI gives a total score of 2–36 m with a cut-off score of 26 differentiating women with and without sexual dysfunction [55, 56]. Lower scores indicate poorer sexual functioning. The validity of the FSFI is not documented [57]. However, the Norwegian version of the FSFI has been shown to have good reliability [58] and is a recommended tool for assessing sexual function by the Norwegian Institute of Public Health [59]. We use a translated Norwegian version of the FSFI.

#### The Stressful Life Events Screening Questionnaire (SLESQ)

SLESQ is a 13-item self-report measure assessing lifetime exposure to traumatic events. Eleven specific and two general categories of events, such as a life-threatening accident, physical and sexual abuse, and witness to another person being killed or assaulted, are examined. For each event, respondents are asked to indicate whether the event occurred (“yes” or “no”), their age at time of the event, as well as other specific items related to the event, such as the frequency, duration, whether anyone died, or was hospitalized. SLESQ has been found to demonstrate satisfactory validity [60]. We use a translated Norwegian version.

### Other data collected

#### Resilience Scale for Adults (RSA)

The RSA is a 33-item self-report scale for measuring protective factors among adults with questions like “I believe in my own abilities”. It has a 7-point response scale with higher scores indicating better protection. The RSA encompasses six factors that cover intrapersonal (e.g., positive perception of self and of future) and interpersonal (i.e., family cohesion and social resources) protective factors presumed to facilitate adaptation to psychosocial adversities. The RSA is developed and tested in Norway and in many other countries. The reliability and validity are good [61].

#### Tonic Immobility Scale

TIS is a two-part, self-report measure designed to assess the presence and severity of specific features of tonic immobility in survivor of sexual victimization. The first part is a 12-item questionnaire, ten of them are used to assess immobility during a sexual assault. Questions like “rate the degree to which you froze or felt paralyzed during your most recent experience” are scored using a 7-point Likert scale ranging from 0 to 6, resulting in a total score between 0 and 72. Higher scores in response to 10 items on the first part of the TIS reflects greater TI behaviors (item 5 and 12 are not part of the total score) [62]. Items contained in part two are considered exploratory and are not used in this study. The validity of the TIS is not documented. We use a translated Norwegian version for gathering information about TI reactions during the sexual assault.

#### Biological measures

We collect samples of the steroid cortisol from patients’ saliva and hair. The saliva cortisol samples are collected 3 days in a row at three specific time points during the day, altogether nine samples, at their morning wakeup, after 30 min and at night (the patient marks the exact time point on the tube tag). Test tubes (SARSTEDT Salivette® Cortisol) containing a sponge for saliva absorption are handed out with oral and written user instructions from the RA to the patients. The patients are instructed to keep the samples in the refrigerator as cortisol in uncentrifuged salivary samples has been shown to be stable for up to 7 days in refrigerated conditions with an allowed bias of 10% and total error allowed of 22% [63]. The collected samples are submitted to the Biobank1®, Trondheim for preparation, registration in BIOBYTE® (de-identification, secure storage of data and material, solution for tracing), and storage of material at minus 80 °C. Later, the samples will be analyzed collectively at the Department of Medical Biochemistry at the St. Olav hospital, Trondheim. Saliva cortisol analysis will be performed using liquid chromatography coupled with tandem mass spectrometry (LCMS/MS) [64]. To reduce systematic analytical variation to a minimum, we will analyze samples collected from the same individuals or from matched individuals collectively, since the samples are collected at different time points and are stored at different conditions before being taken care of by the Biobank1®.

A small hair sample (strands of 3 mm in diameter) is collected from the patient, cutting as close as possible to the scalp from a posterior vertex position, and packed into a folded aluminum foil for storage at the Biobank1®, Trondheim together with the saliva cortisol samples. Measurements of cortisol in hair will later collectively be sent for analyses at the laboratory of Prof. Dr. Clemens Kirschbaum, Technische Universität Dresden, Germany. Hair steroid analysis will be performed using the LCMS/MS method [65]. The hair samples are additionally collected as these measurements provide a retrospective indication of the cumulative cortisol secretion over an extended period, which represents a good proxy indicator of the intraindividual stability regarding cortisol levels [66]. The hair and the saliva cortisol collections thus represent trait versus state-dependent measurements of circulating stress hormone.

#### Physical activity and sleep measures

Physical activity and sleep will be objectively measured using two AX3 (Axivity, Ltd., UK) accelerometers attached to the skin at the right thigh and lower back. The sensor streams are analyzed using a machine learning model, providing an overall accuracy of about 95% in detecting total sleep time (TST), movements per time unit (indicator of fragmented sleep), and time spent sitting, standing, walking, running, cycling, and lying down [67, 68].

### Medical record data and practicalities in web-CRF

To reduce stress and workload on the patients, the research coordinators on each study site (SAC) will collect medical record data regarding the assault, as well as relevant clinical information. Data are plotted into a web-based data collection system (web-CRF3) developed and administered by the Clinical Research Unit, NTNU/St. Olavs hospital. Through this process, all information is de-identified and encrypted.

The RA will enter practical monitoring data into the same web-CRF3 system.

Similarly, all steps performed in the intervention mPE and in TAU will be collected by the nurses/social workers performing the treatment in the same web-CRF3 system.

### Statistical analyses

The main statistical analysis for testing the primary and secondary hypotheses will be generalized linear mixed model (GenLinMixed) following an intention-to-treat principle. The primary analysis strategy is planned as an analysis of covariance method with three fixed factors, including main effect for Group (mPE + TAU vs TAU differences), a main effect for Time (allowing different scores across the repeated post-intervention measures), and an interaction Group*Time (allowing for different treatment effects at different post-intervention time points). The treatment effects at 6 weeks and at follow-up 3, 6, and 12 months later will then be estimated using four planned comparison tests. An alpha level of < 0.05 is required for the primary hypothesis tests but lowered to < 0.01 for the secondary analyses due to the increased number of tests. The baseline value of the outcome will be included as a covariate in these analyses as this increases statistical power [69], and other baseline variables which are predictive of the outcome will be considered for inclusion. The analysis of covariance method is preferred in terms of less bias and better precision, and ultimately, statistical power [70]. The exploratory moderator analyses will extend the above regression models by adding the moderator variable in question, together with its two-way interactions with Time and Group and the full three-way interaction in order to discern whether moderator effects only show up at specific time points in one of the two study arms. The GLMM approach is also favorable regarding the handling of missing data as this provides unbiased estimates under the assumption that data are missing at random (MAR). In the case of a high drop-out rate after the baseline measurements, we will consider an alternative repeated measure GLMM model where the baseline score is included as one of the repeated outcome measures, rather than as a covariate, as recommended by Twisk et al. (equation 2c) [71]. The GLMM method is also highly flexible in handling dependencies in the data, which is expected for the repeated measures of each individual (level 1), as well as for the three study sites (level 2), and potentially also for patients nested within therapist effects. The analysis strategy will be described in detail in a separate Statistical Analysis Plan (SAP) which will be finalized prior to unblinding the data.

### Power and sample size calculation

Since the randomization occurs within each study site, the statistical power is estimated using the blocked individual random assignment procedure of PowerUp in Studio R [72]. We anticipate that approximately 185 patients will be eligible and consent to participate (from the Oslo SAC, *n* = 110, from the Trondheim SAC,* n* = 55, and from the Vestfold SAC, *n* = 20), of which approximately 85 (46%) will drop out during the study period. Given a final sample size of 100 completed participants by the 3 months’ assessment, presuming an alpha = 0.05, a two-tailed test, three study sites at level 2, and a baseline-outcome correlation of 0.60 (*R*-square = 0.36), we have an 80% power to detect a standardized mean difference (SMD) of minimum 0.44 for each of the primary outcomes. In practical terms, an average reduction of 0.44 standard deviation in the PCL-5 score would mean that an expected 36.2% of sexually assaulted victims risk developing PTSD without intervention (according to Dworkin [5]), whereas 21.4% of those who receive the intervention would be expected to develop PTSD due to the shift in the distribution of scores.

## Oversight and monitoring

### Data management and storage

All patients recruited into the study will get a unique participant’s ID number. The ID number, which is linked to the patient’s personal 11-digit ID, will be stored in a separate research file area provided by the Services of sensitive data (TSD) at the University of Oslo, that is stored with highest possible level of security (two-factor digital identification system), and in data file areas with continuous back-up systems. Only the principal investigators have access to these areas. Data will be transformed into separate Excel, SPSS, or STATA files in the TSD system to be used for statistical analyses. Trial data are stored until the completion of analysis (data collection estimated to be finished by Dec 31, 2025), then deleted after at least 5 years.

For data collected through web-CRF3 and eFORSK, we will use the secure transfer system developed by the Clinical Research Unit at the NTNU/St. Olavs hospital. Identifiable data will be downloaded from encrypted files and stored in separate secure research file areas provided from the Data Protection Official at the St. Olavs hospital. Identifiable data will be downloaded from encrypted files and stored in separate secure research file areas provided from the Data Protection Official at St. Olavs hospital. De-identified working files will be transferred into TSD for analyses.

The collected biological material (saliva and hair samples) is submitted identifiable to the Biobank1®, Trondheim, who will register time points for sample collection, preparation, and freezing in the BIOBYTE® system. The identifiable personal 11-digit ID is encrypted and de-identified when material is stored. However, a solution is kept for tracing, in order to be able to connect to other study data later on.

A Data Management Plan for this project has been published at the Norwegian Centre for Research Data (NSD). A Data Protection Impact Assessment (DPIA) with risk and security measures has been published at the St. Olavs hospital (sponsor’s) internal websites and the Data Protection Official at the St. Olavs hospital and at the NTNU have both acknowledged our DPIA.

### Trial steering committee

The trial steering committee consists of the project management (PM); Hagemann, Halvorsen, Schei, and Ellen Bugge, Senior advisor at the NGO the Norwegian Women’s Public Health Association (N.K.S.), and in addition, Elisabeth T. Swärd, Senior advisor on women’s health and research, the N.K.S., and Tone Shetelig Løvvik, the Head of the Department of Obstetrics and Gynecology, St. Olavs hospital, Trondheim, Norway. The decision-making body of the study consists of the principal investigator (PI) CH, associate investigators JØH, OF, and the Ph.D. candidate TH. The research group collaborate on a daily basis with all other working parts of the study (e.g., research assistants and research coordinators at each site) to maintain compliance, recruitment, and treatment fidelity.

### Data monitoring committee (DMC)

The data monitoring committee (DMC) will review the safety aspects of the trial and the validity and integrity of the collected data. Members are Øyvind Salvesen, statistician and associate professor at the Faculty of Medicine and Health Sciences, NTNU; Katrine Høyer Holgersen, Clinical psychologist and associate professor at the Department of Psychology, NTNU; and Ida Heiaas, Senior project advisor at the N.K.S. All members are independent of the research group members (the PM and Steering committee) and have no conflict of interest with the research group. The DMC will evaluate adverse events (reported through web-CRF3), revisions, and deviations of the protocol. All changes and modifications have been and will be communicated to REC for approval according to the contract and updated in the clinical trial registry. Any deviations from the protocol will be fully documented using a breach report form.

### Safety and monitoring procedures of adverse events

Procedures are in place to monitor adverse and serious adverse events (AEs and SAEs) that participants may experience during the trial. The SAC staff are instructed to fill out case reports (in the web CRF3) of any AEs and SAEs occurring during the trial period. AEs are defined as any undesirable experience occurring to participants during the trial, and they are categorized as to whether they are perceived as related to the interventions or not, expected or not, and if any treatment was necessary. SAEs are defined as death, suicide, attempted suicide, serious self-harm, acute psychosis or mania, severe intoxication, or other psychological or somatic illnesses or conditions that demand acute treatment (epilepsy, heart attack, stroke). Members of the research group will be alerted automatically by e-mail within 24 h if an AE or SAE is registered.

## Dissemination

The results of the study will be disseminated in scientific journals regardless of the results.

## Discussion

The main aim of the present trial is to investigate whether mPE provided shortly after rape or attempted rape as an add-on to treatment as usual is more effective than treatment as usual in preventing the development of posttraumatic sequelae. Rape and sexual assault are the traumatic life event that carries the highest conditional risk for the development of PTSD. Victims of sexual assault are generally considered vulnerable but not as having a condition or disorder that require intervention and are therefore seldom subject to intervention trials. Another reason for the lack of early intervention studies may be due to existing treatment guidelines recommending “watchful waiting” until symptoms of an underlying PTSD become more evident, possibly also interfering with natural recovery following such a traumatic event. Early clinical studies by Rothbaum et al. repel these concerns and show that early behavioral interventions are safe and efficacious [22]. In addition, a Danish study of 51 victims of sexual assault indicated that most women evaluated their participation in research as a positive experience causing them little psychological or physiological distress [73].

Given the support of the hypotheses, i.e., that mPE may prevent development of PTSD in women exposed to sexual assault and potentially also preventing subsequent comorbid health problems (e.g., sexual dysfunction), the trial will provide new knowledge regarding the benefits of implementing preventive measures as part of the health service portfolio of the Norwegian SACs. In connection with the planned moderator analyses, the study may yield additional insight into which subgroups of women that may benefit the most, or conversely, the least, from receiving this kind of psychological intervention. The knowledge generated from the project offers the first-line health care services a better foundation for knowing which individuals are particularly vulnerable to develop chronic PTSD and for providing women more efficient or better tailored care. In the end, the project may contribute to the revision of guideline related to the psychosocial procedures and professional treatment and care that the SACs offer.

Some important secondary aims relate to whether preventive initiatives as planned here also may prevent sexual dysfunctions, pelvic floor hyperactivity, and chronic pelvic and vulvar pain. It may also evaluate whether prevention of PTSD symptoms through the mPE intervention may be a clinical mechanism (i.e., a mediator) responsible for reducing pelvic floor hyperactivity and sexual dysfunction. In addition, mPE may facilitate regulation of the endocrine stress systems (measured through saliva and hair cortisol), and the study results may provide insights into the potential for combined drug treatment with cortisol to sustain a healthy adaptation.

### Strengths and limitations

Major strengths of the study are the multi-site recruitment and the use of SAC nurses, social workers, and other health professionals at the centers as therapists responsible for delivering the intervention. Studies on disseminating PE from the specialist clinics to other contexts (e.g., other social communities) have shown that non-experts in CBT (cognitive behavioral therapy) when supervised and trained by CBT-experts can deliver PE efficiently [74]. If an evidence-based intervention such as mPE can be implemented in the SACs and delivered by nurses and social workers, this could improve the generalizability of the findings considerably as this implementation mimics the normal operation within the SACs. The traditional use of adherence checks helps clarify to what extent the nurses/health personnel deliver the mPE protocol as trained to do.

We use a strong methodological approach, mPE, as an addition to the ordinary services as provided by the SAC, i.e., TAU. This implies that the mPE study arm receives much of the same services as the TAU study arm, except they receive mPE in addition, which to a large extent rules out any inherent placebo effects. Thus, if the mPE do better than TAU by dismissing the null hypothesis of equal intervention effects, the support of mPE as superior to TAU is strong given then randomized clinical design.

Recruitment may be hampered if too many women rather prefer to suppress than learn to actively manage trauma reminders or trauma memories they are struggling with.

The intervention is not blinded, neither to the patients, nor to the SAC staff; hence, the assessments could be affected by this (e.g., those not getting mPE could want to drop out more easily than the mPE group).

The assessments could be too much of a workload for the participating patients, thereby hindering recruitment; therefore, we have decided that only the questionnaire’s PCL-5 and the EQ-5D-5L, as well as the PSS-I-5 interview, is mandatory to fulfill at each assessment. Hence, there could be missing information on less crucial questionnaires, hair and saliva cortisol, and activity measures to a larger degree than originally wanted.

### Trial status

Recruitment has started in June 2022 and is still ongoing. The estimated completion date is Dec 31, 2025.

### Supplementary Information

**Additional file 1. **SPIRIT checklist.

## Data Availability

Anonymized and analyzed data and statistical codes will be available from the corresponding author on reasonable request, as is the full protocol.

## Abbreviations

PTSD: Posttraumatic stress disorder
mPE: Modified prolonged exposure
SAC: Sexual assault center
TAU: Treatment as usual
EMDR: Eye Movement and Desensitization Reprocessing
DSM-5: Diagnostic and Statistical Manual for mental disorders, fifth edition
SPIRIT: Standard Protocol Items: Recommendations for Interventional Trials
RA: Research assistant
PSS-I-5: PTSD Symptom Scale Interview for DSM-5
CRF: Case report form
PE: Prolonged exposure
ICC: Intraclass correlation coefficient
PCL-5: PTSD Symptom Checklist for DSM-5
PHQ-9: Patient Health Questionnaire-9
GAD-7: General Anxiety Disorder-7
EQ5D5L: EuroQol 5L health-related quality of life
BIS: Bergen Insomnia Scale
AHPFS-W: Amsterdam Hyperactive Pelvic Floor Scale for Women
FSFI: Female Sexual Function Index
SLESQ: Stressful Life Event Screening Questionnaire
TIS: Tonic Immobility Scale
RSA: Resilience Scale for Adults
AE: Adverse events
SAE: Serious adverse events
GAD: General Anxiety Disorder
LCMS/MS: Liquid Chromatography coupled with tandem Mass Spectrometry
AX3: 3 Axis Accelerometer
TST: Total sleep time
GLMM: Generalized linear mixed models
MAR: Missing at random
SAP: Statistical analysis plan
SMD: Standard mean difference
TSD: Tjenester for Sensitive Data (Services for sensitive data)
NSD: Norsk Senter for Forskningsdata (Norwegian Center for Research Data)
DPIA: Data Protection Impact Assessment
PM: Project Management
NGO: Non-governmental organization
NKD: Norske Kvinners Sanitetsforening (Norwegian womans public health association)
PI: Principal Investigator
DMC: Data Monitoring Committee
REK-Midt: Regional Etisk Komite (Regional committee for medical and health research ethics)
GCP: Good Clinical Practice
CBT: Cognitive behavioral therapy
NORCE: Norwegian Research Center
