# Correction: Modified prolonged exposure therapy as Early Intervention after Rape (The EIR-study): study protocol for a multicenter randomized add-on superiority trial

**DOI:** 10.1186/s13063-023-07585-6

**Published:** 2023-10-03

**Authors:** Tina Haugen, Joar Øveraas Halvorsen, Oddgeir Friborg, Melanie Rae Simpson, Paul Jarle Mork, Gustav Mikkelsen, Ask Elklit, Barbara O. Rothbaum, Berit Schei, Cecilie Hagemann

**Affiliations:** 1 Department of Psychology, Norwegian University of Science and Technology (NTNU), NO-7491 Trondheim, Norway; 2 Department of Clinical and Molecular Medicine, Norwegian University of Science and Technology (NTNU), NO-7491 Trondheim, Norway; 3 St. Olavs Hospital, Trondheim University Hospital, Pb. 3250 Torgarden, 7006 Trondheim, Norway; 4 Department of Psychology, The Arctic University of Norway (UiT), Pb. 6050 Langnes, N-9037 Tromsø, Norway; 5 Department of Public Health and Nursing, Norwegian University of Science and Technology (NTNU), Pb. 8905, N‑7491 Trondheim, Norway; 6 Department of Clinical Chemistry, St. Olavs Hospital, Trondheim University Hospital, Pb. 3250 Torgarden, 7006 Trondheim, Norway; 7 National Danish Center for Psychotraumatology, University of Southern Denmark, Campusvej 55, 5230 Odense, Denmark; 8 Department of Psychiatry, Veterans Program and the Trauma and Anxiety Recovery Program, Emory University School of Medicine, Atlanta, USA; 9 Department of Obstetrics and Gynecology, St. Olavs Hospital, Trondheim University Hospital, Pb. 3250 Sluppen, NO‑7006 Trondheim, Norway

**Correction: BMC Trials 24, 126 (2021)**

**https://doi.org/10.1186/s13063-023-07147-w**

Following publication of the original article [1], we have been informed that the box entitled “Baseline assessments” in Fig. 1 was misplaced. The correct placement should have been after “Informed consent” and before “Randomization”.

Originally published figure:
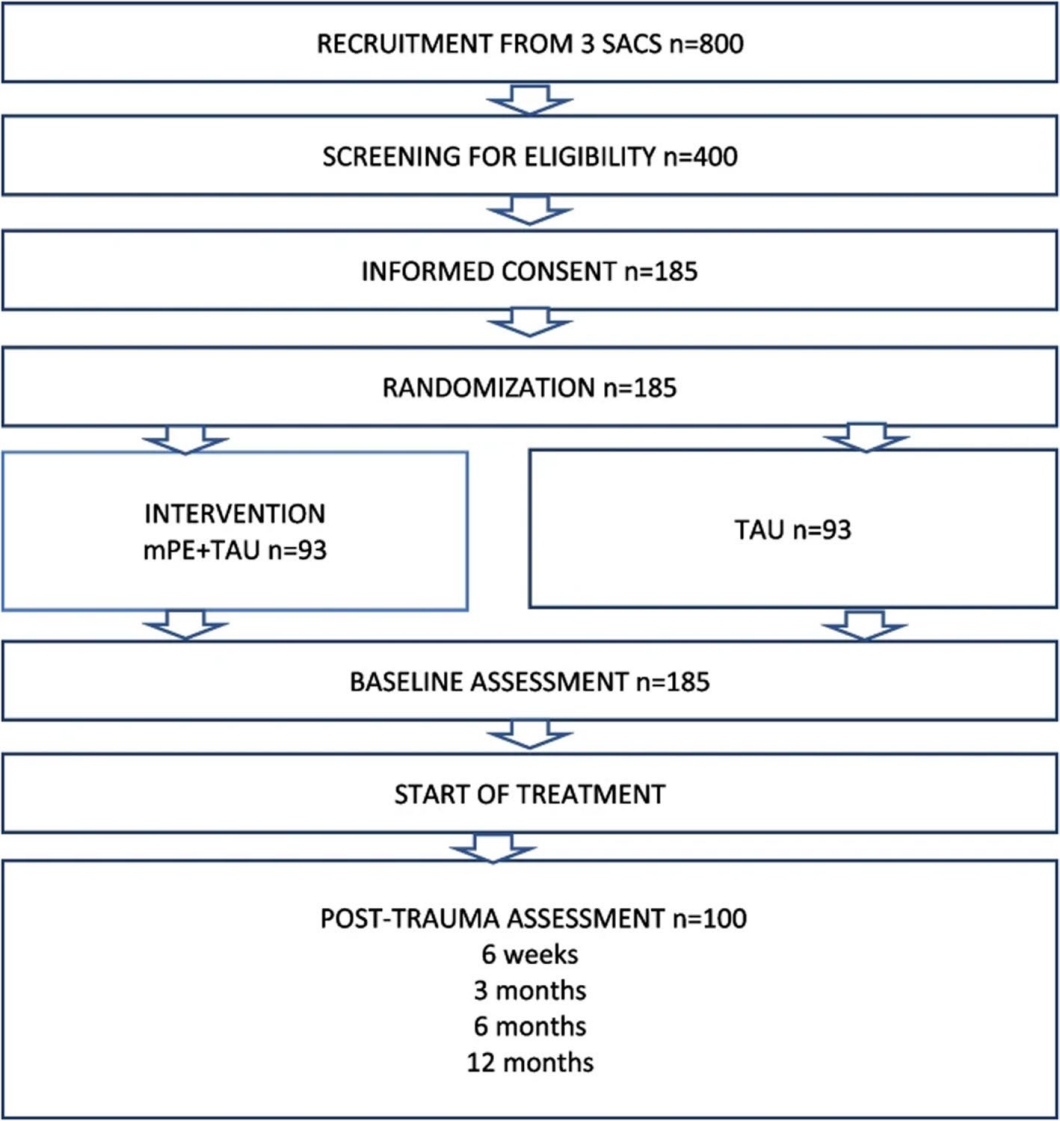


Corrected figure:
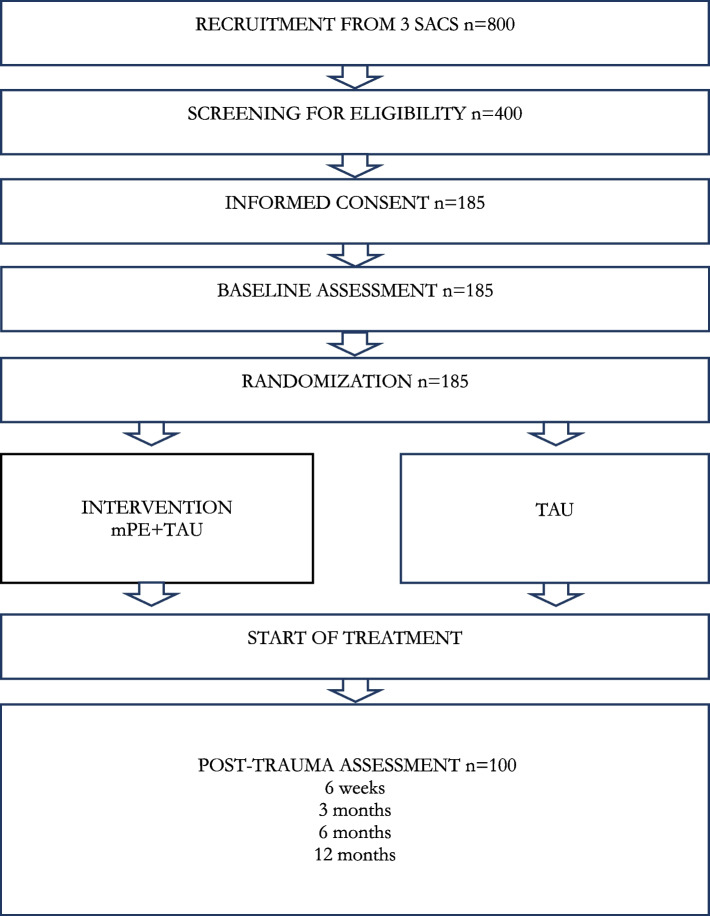


The original article has been corrected.
